# The 12-Item Self-Rating Questionnaire for Depressive Mixed State (DMX-12) for Screening of Mixed Depression and Mixed Features

**DOI:** 10.3390/brainsci10100678

**Published:** 2020-09-27

**Authors:** Hotaka Shinzato, Yu Zamami, Tsuyoshi Kondo

**Affiliations:** Department of Neuropsychiatry, Graduate School of Medicine, University of the Ryukyus, Okinawa 903-0215, Japan; zam.zam.yu.pyon@gmail.com (Y.Z.); kondo@med.u-ryukyu.ac.jp (T.K.)

**Keywords:** depressive mixed state, screening, DMX-12, receiver operating characteristic

## Abstract

For simultaneous screening of mixed features (MF) by DSM-5 and mixed depression (MD) by Benazzi, useful symptoms were extracted from our 12-item dimensional scale for depressive mixed state (DMX-12). Subjects were 190 consecutive cases with major depressive episode (MDE) who visited our clinic. Associations between symptomatological combinations of the DMX-12 and MF or MD were analyzed using receiver operating characteristic (ROC). The rate of MF was 4.2% while that of MD was 22.6%. Eight symptoms (overreactivity, inner tension, racing/crowded thought, impulsivity, irritability, aggression, risk-taking behavior, and dysphoria) with their AUC > 0.6 for ROC curves were specially focused on distinguishing patients with MF or MD from non-mixed patients. By using these 8 symptoms, 40.5% of the overall patients were screened as positive at the same cut-off value (≥13) for both MD and MF. The AUC of ROC curve and sensitivity/specificity were well balanced together with sufficient negative predictive values. The abovementioned 8 symptoms seem to be helpful for primary screening and negative check of DMX with considerable severity during MDE.

## 1. Introduction

Depressive mixed state (DMX) as a mixture of subthreshold manic components during major depressive episodes (MDE) [1] remains underdiagnosed although clinicians do not rarely encounter potential DMX in usual clinical settings. Core Symptoms of DMX have been regarded as distractibility, psychomotor agitation, irritability, and racing/crowded thoughts, which are apparently different from static and internalized manifestations of pure depression [2]. However, patients rarely express their mixed depressive symptoms, whereas clinicians tend to focus on typical depressive manifestations and overlook DMX during MDE [3].

Moreover, DMX often requires prompt treatment including urgent admission and cautious medication different from those used for pure depression like vigorous antidepressant monotherapy [3,4].

Mixed episode used to be a rare diagnosis only for bipolar I disorder, because the DSM-IV-TR [5] criteria defined mixed episode as a mixture of both full depressive and full manic episodes. The new diagnostic criteria, DSM-5, more broadly defined DMX as “mixed features specifier (MF)”, at least three typical hypomanic/manic symptoms during a major depressive episode [6]. However, contrary to our expectations, studies using the DSM-5 criteria [6] used to demonstrate relatively low prevalence of MF (3.2–7.5%) [7,8] or rather show considerably wide range of the prevalence according to the latest meta-analysis (7.2–42.5%) [9].

Other studies [10,11,12] have criticized that exclusion of nonspecific symptoms like distractibility, irritability, and agitation common for both manic and depressive psychopathology causes underdiagnosis of MF and have stressed that such overlapping symptoms are rather important as the core symptoms of DMX. “Mixed depression (MD)” proposed by Benazzi has covered these excluded nonspecific symptoms as core mixed symptoms and has extended the definition of DMX (at least 3 mixed symptoms for a week and more during MDE) [13,14,15,16].

When the diagnostic criterion for MD was applied to depressed patients, the prevalence of DMX was estimated as about one-third of MDE, which was 5 to 10 times higher than that diagnosed by MF of the DSM-5 criteria [8] Thus, categorically diagnosed DMX has been still a target of argument, because of large variability in their prevalence without any assessment of DMX severity.

Recently, we proposed the importance of dimensional assessment of DMX [3] and developed the 12-item questionnaire for quantification of depressive mixed state (DMX-12) as a severity assessment of clinically relevant DMX, which covered nonspecific but frequent mixed symptoms [17]. Exploratory factor analysis revealed that the DMX-12 consisted of such 3 clusters as spontaneous instability, vulnerable responsiveness, and disruptive emotion/behavior [17].

Although we also referred to its plausible usefulness of the DMX-12 as a screening tool for DMX [17], ROC analysis had not been performed, thereby, it was still uncertain whether the DMX-12 was clinically helpful for screening of DMX or not. Therefore, in the present study, we aimed to clarify the practical usefulness of various combinations of the DMX-12 symptoms with the best cut-off for screening of clinically relevant DMX.

## 2. Subjects and Methods

### 2.1. Subjects

This study was conducted during the period between June 2014 and December 2019. Subjects were consecutive 190 patients, who visited our outpatient psychiatry clinic of University of the Ryukyus Hospital and were diagnosed as having MDE during the aforementioned period. Two experienced psychiatrists confirmed MDE according to the DSM-5 diagnostic criteria [6] and further classified MDE into unipolar depression and bipolar and related disorders. Patients with alcohol use disorder or dementia were excluded from analyses.

All subjects gave written informed consent to participate in the study (when patients were under the age of 18, consent was also obtained from their parents). We provided explanation of the purpose of this survey, the right to withdraw from the analysis, no medical inconvenience to refuse and protection of personal information to each participant. The data were dealt with as anonymous and were coded for group analyses. 

This study protocol was conducted in accordance with the Declaration of Helsinki. The study protocol was approved by the Ethics Committee of University of the Ryukyus for Medical and Health Research Involving Human Subjects on 5 June 2014 with approval number 658, and was re-approved on 1 May 2017 with approval number 658 (change 1) due to changes in Japanese law regarding clinical research.

### 2.2. Assessments and Statistics

**Figure A1:**
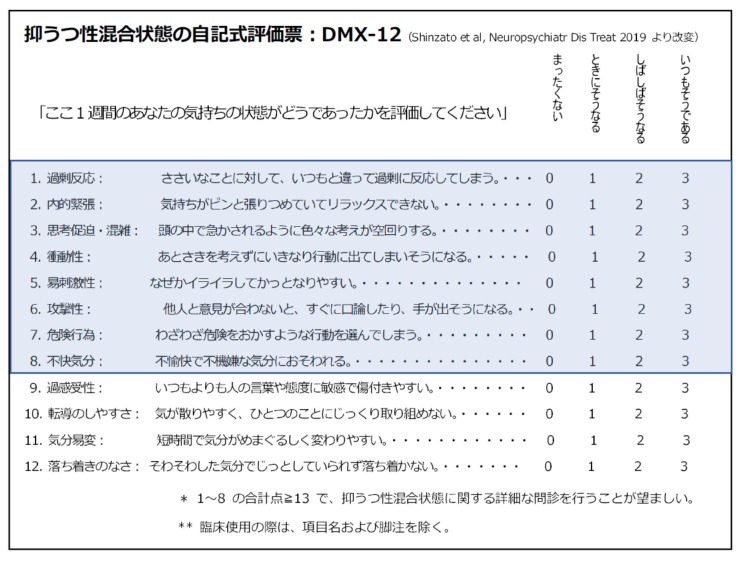
The 12-item questionnaire for quantification of depressive mixed state in Japanese.

**Figure A2:**
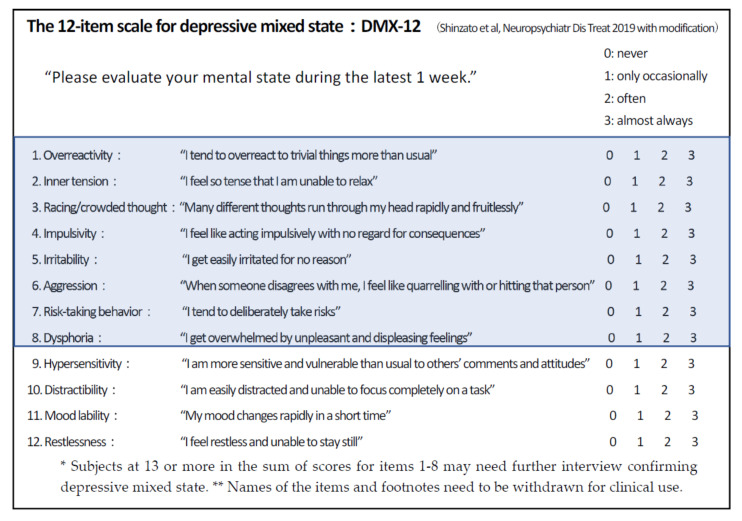
The 12-item questionnaire for quantification of depressive mixed state in English.

The original DMX-12 in Japanese was developed by us (Appendix A Figure A1) and was also translated into English by two native speakers (Appendix A Figure A2) [17]. The 12 symptoms were hypersensitivity, overreactivity, distractibility, mood lability, inner tension, dysphoria, racing/crowded thought, restlessness, impulsivity, irritability, aggression, and risk-taking behavior, which had ever been suggested as core DMX symptoms in previous studies [3,7,15,18]. Each item of the DMX-12 was scored by four-scale steps (0: never, 1: only occasionally, 2: often, 3: almost always) according to its frequency in the latest week (Appendix A Figure A1 and Figure A2). In the present study, we used the original version of the DMX-12 in Japanese.

MD was diagnosed according to the Benazzi’ definition of MD [13,14,15] while the diagnosis of MF was made by the criteria for “depressive episodes, with mixed features” according to DSM-5 [6]. These were adopted as the representative for categorical diagnoses of DMX to investigate their relationships with dimensional assessment of DMX by the DMX-12. Comparisons of clinical backgrounds between non-mixed patients and patients with MD or MF were performed by Mann–Whitney U-test and chi-square test or Fisher’s exact test. Total DMX-12 score and its subscale scores were compared between individuals with and without categorically diagnosed DMX by Mann–Whitney U-test. The optimal cut-off values of several combinations of the DMX-12 symptoms to identify MD or MF were analyzed by using receiver operating characteristic (ROC) curves with the maximal Youden Index as a probe. A two-tailed *p-*value less than 0.05 was considered statistically significant. EZR software version 1.27 (Saitama Medical Center, Jichi Medical University, Saitama, Japan) [19] was used for all statistical analyses.

## 3. Results

The 190 subjects consisted of 72 males and 118 females. The mean age (±SD) was 43.5 (±17.7), and the age range was from 13 to 85 (teens: 16, twenties: 30, thirties: 36, forties: 40, fifties: 29, sixties: 23, and seventies or more: 16). The 138 patients were diagnosed as unipolar depression while 52 patients were diagnosed as bipolar and related disorders.

Among the 190 patients with MDE, 43 cases (22.6%) fulfilled Benazzi’s criterion for MD [13,14,15] while 8 cases (4.2%) fulfilled the DSM-5 criteria for MF [6]. Demographic data for patients with MD and MF are summarized in Table 1. Patients with MD or MF had more mood episodes and bipolarity than non-mixed patients (Table 1). Significant differences were found in total scores of the DMX-12 between MD and non-MD patients (22.9 ± 5.8 versus 17.0 ± 8.0, *p* = 0.001) or between MF and non-MF patients (24.4 ± 7.7 versus 18.1±7.9, *p* = 0.034), as shown in Table 1. Among the 3 clusters of the DMX-12, only the “disruptive emotion/behavior” subscale significantly differentiated both MD from non-MD (6.0 ± 2.6 versus 3.6 ± 2.8, *p* = 0.001) and MF from non-MF (7.1 ± 2.6 versus 4.0 ± 2.8, *p* = 0.005), which was consistent with our previous finding [17]. Thus, the subscales “spontaneous instability” and “vulnerable responsiveness” were excluded from target syndromes for ROC analyses.

Values of AUC for ROC curves by the DMX-12 symptoms are shown in Table 2. Overreactivity, inner tension, racing/crowded thought, impulsivity, irritability, aggression, risk-taking behavior, and dysphoria showed AUC values more than 0.6 for their ROC curves differentiating both MD and MF from non-mixed state (Table 2). Accordingly, ROC analysis was also made by using total scores of these 8 symptoms.

**Figure 1:**
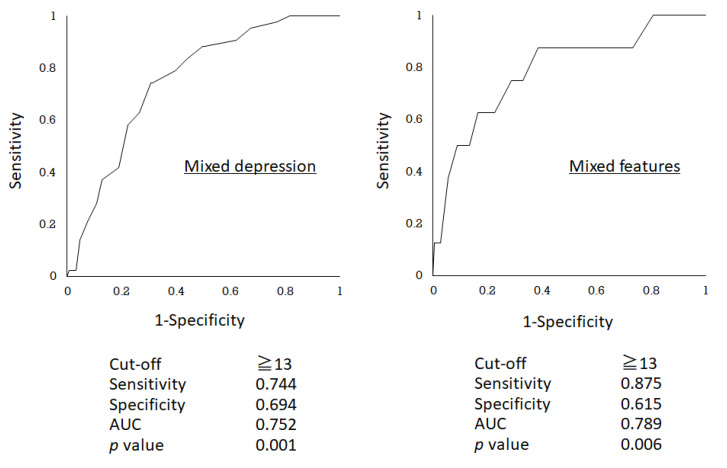
Receiver operating characteristic (ROC) curves determining mixed depression or mixed features by selective 8 mixed symptoms (overreactivity, inner tension, racing/crowded thought, impulsivity, irritability, aggression, risk-taking behavior, and dysphoria).

By using these 8 symptoms, 40.5% of the overall patients (33.3% for depressive disorders and 59.6% for bipolar and related disorders) were screened as positive at the same cut off value (≥13) for both MD and MF (Table 3). The AUC of ROC curve (0.752 for MD and 0.789 for MF) and sensitivity/specificity for screening (0.744/0.694 for MD and 0.875/0.615 for MF) using the selected 8 symptoms were well balanced together with sufficient negative predictive values (0.903 for MD and 0.991 for MF) compared with those using total DMX-12 or its disruptive emotion/behavior subscale (Table 3 and Figure 1).

The original version of the DMX-12 questionnaire [17] was then rearranged according to the abovementioned advantage of use of the selected 8 symptoms in distinguishing DMX patients from non-DMX patients using the appropriate cut-off. (Appendix A Figure A1 and Figure A2).

## 4. Discussion

The conventional criteria for categorical diagnoses of DMX have left controversial problems, i.e., potential overdiagnosis of DMX by using MD criterion and probable underdiagnosis of DMX by using MF criteria [7,8]. We have also suggested that the categorical diagnoses of DMX have limitations due to potential concern for both aspects of overdiagnosis and underdiagnosis and lack of severity assessment of DMX [3]. In fact, a recent review has implied that DMX has a dimensional aspect rather than a categorically-defined concept [11]

In recent years, various evaluation scales with dimensional approaches to DMX diagnosis have been developed to eliminate aforementioned concerns [20,21,22,23]. However, these scales have some difficulties for routine clinical use, e.g., inclusion of infrequent mixed symptoms, time-consuming process, and necessity of objective assessments, which may not necessarily be suitable for screening tool of DMX in primary care settings.

The DMX-12 is a simple and easy scale that can be self-evaluated by patients themselves, consisting of 12 non-specific but frequent mixed symptoms as a dimensional assessment of DMX [17]. The DMX-12 was originally thought to be useful in assessing severity of DMX and determining response to its treatment. Meanwhile, this scale was also expected as a screening tool of treatment-requiring DMX with considerable severity [17].

Among 3 subscales of the DMX-12, only the “disruptive emotion/behavior” cluster was closely associated with MD and MF. The “disruptive emotion/behavior” subscale appears to provide specific manifestations but may not be sensitive enough to link with various types of DMX, because it only covers 4 apparent mixed symptoms. Therefore, we decided to seek more promising combinations of the DMX-12 symptoms for a useful screening of DMX by conducting ROC analyses comprehensively.

As a result, the 8 symptoms (overreactivity, inner tension, racing/crowded thought, impulsivity, irritability, aggression, risk-taking behavior, and dysphoria) out of the DMX-12 symptoms showed more than 0.6 in the AUC values of ROC differentiating both MD and MF from non-mixed state (Table 2). In fact, the sum of these 8 symptoms scores showed the best sensitivity/specificity balance together with excellent negative predictive value, compared with other combinations of the DMX-12 symptoms (Table 3 and Figure 1). Furthermore, it coincidentally distinguished both MD from non-MD and MF from non-MF, by using the same cut-off score at 13, resulting in reasonable ratio for screening as positive (40.5%) out of the overall patients (Table 3 and Figure 1). Based on efficient screening for both MD and MF, well-balanced sensitivity/specificity profile and sufficient negative predictive value together with convenience to use the same cut-off for different categorical diagnoses of DMX (Table 3), it is suggested that the selected 8 items may be the most useful as primary screening and negative check of DMX.

Our previous study has warned that careful attention should be paid on differential diagnosis of DMX during MDE from borderline personality disorder since the disruptive emotion/behavior symptoms of the DMX-12 are sometimes externalized as interpersonal frictions and dangerous acting out as can be similarly seen in patients with borderline personality disorder [17]. Nonspecific mixed symptoms of the DMX-12 cannot differentiate cross-sectional psychopathology between DMX and borderline personality disorder whereas core feature of typical hypomania was not related to borderline personality trait [24]. Thus, the differential diagnosis should be rather based on the differentiation between state (DMX) and trait (borderline personality) which longitudinal course observation may reveal (i.e., recurrence/switch of mood episodes in contrast to stabilized emotional control and interpersonal relationship during remitted periods in DMX patients). Other than disease course, family history of bipolarity and treatment response to medication may be helpful for the differential diagnosis [25].

This study has some other limitations. First, subjective bias like exaggeration or underestimation of DMX may accompany self-rating scale by patients. Second, scoring was not made by descriptive severity, but simply by frequency of DMX symptoms. Third, reliability and validity of the DMX-12 in English version have not been examined yet.

Despite the limitations above, it should be noted that the 8 symptoms selected from the DMX-12 may be clinically efficacious in screening treatment-requiring DMX with considerable severity in a busy clinical setting, efficiently followed by further diagnostic interviewing for definite diagnosis of DMX.

## 5. Conclusions

Eight symptoms (overreactivity, inner tension, racing/crowded thought, impulsivity, irritability, aggression, risk-taking behavior, and dysphoria) selected from the DMX-12 are helpful for primary screening and negative check of clinically relevant DMX with considerable severity, in accordance with conventional categorical diagnoses such as MD and MF.

## Figures and Tables

**Table 1:** Comparisons of clinical backgrounds between non-mixed patients and those with mixed depression or mixed features.

	Mixed Depression			Mixed Features		
	Present (*n* = 43)	Absent (*n* = 147)	*p*	Present (*n* = 8)	Absent (*n* = 182)	*p*
Age (years)	40.1 ± 17.3	44.6 ± 17.8	0.121	43.5 ± 20.7	43.5 ± 17.7	0.885
Female gender (%)	67.4%	60.5%	0.412	62.5%	62.1%	1.000
Number of mood episodes	4.0 ± 3.5	2.9 ± 2.9	0.022	6.3 ± 4.1	3.0 ± 2.9	0.012
Duration of illness (year)	7.8 ± 8.6	6.5 ± 7.7	0.265	9.9 ± 7.9	6.7 ± 7.9	0.188
High education level (%)	37.2%	38.8%	0.828	62.5%	37.4%	0.264
Bipolarity (%)	48.8%	21.1%	0.001	75.0%	25.3%	0.006
DMX-12 (total score)	22.9 ± 5.8	17.0 ± 8.0	0.001	24.4 ± 7.7	18.1 ± 7.9	0.034

High education level means graduation from university or upper.

**Table 2:** Values of AUC for ROC curves differentiating mixed depression or mixed features.

	Mixed Depression (MD)	Mixed Features (MF)
***Vulnerable responsiveness***		
Hypersensitivity	0.591	0.410
**→**Overreactivity	0.692	0.665
***Spontaneous instability***		
Distractibility	0.586	0.486
Mood lability	0.581	0.622
Restlessness	0.590	0.644
**→**Inner tension	0.641	0.630
**→**Racing/crowded thought	0.634	0.645
**→**Impulsivity	0.651	0.786
***Disruptive emotion/behavior***		
**→**Irritability	0.645	0.671
**→**Aggression	0.650	0.728
**→**Risk-taking behavior	0.651	0.804
**→**Dysphoria	0.713	0.644

Arrows indicate 8 items with AUC values > 0.6 for ROC curves differentiating both MD and MF.

**Table 3:** Results of ROC analyses using total and cluster scores of the DMX-12.

	Cut-Off (Positive Ratio %)	Sensitivity	Specificity	PPV	NPV	AUC of ROC
**Mixed depression**						
Total score of the DMX-12	≥19 (47.9%)	0.767	0.605	0.363	.899	0.720
4 symptoms of disruptive emotion/behavior subscale	≥4 (50.5%)	0.814	0.585	0.365	0.915	0.741
8 symptoms selected for screening of DMX	≥13 (40.5%)	0.744	0.694	0.416	0.903	0.752
**Mixed features**						
Total score of the DMX-12	≥23 (34.2%)	0.750	0.676	0.092	0.984	0.722
4 symptoms of disruptive emotion/behavior subscale	≥7 (24.7%)	0.750	0.775	0.128	0.986	0.791
8 symptoms selected for screening of DMX	≥13 (40.5%)	0.875	0.615	0.091	0.991	0.789
